# Age-Related Variations in Postoperative Pain Intensity across 10 Surgical Procedures: A Retrospective Study of Five Hospitals in South Korea

**DOI:** 10.3390/jcm12185912

**Published:** 2023-09-12

**Authors:** Jong-Ho Kim, Jong-Hee Sohn, Jae-Jun Lee, Young-Suk Kwon

**Affiliations:** 1 Department of Anesthesiology and Pain Medicine, Chuncheon Sacred Heart Hospital, Hallym University College of Medicine, Chuncheon-si 24253, Republic of Korea; poik99@hallym.or.kr (J.-H.K.); iloveu59@hallym.or.kr (J.-J.L.); 2 Institute of New Frontier Research Team, Hallym University College of Medicine, Chuncheon-si 24252, Republic of Korea; deepfoci@hallym.or.kr; 3 Department of Neurology, Chuncheon Sacred Heart Hospital, Hallym University College of Medicine, Chuncheon 24253, Republic of Korea

**Keywords:** age, pain, surgery, opioid

## Abstract

Age-related differences in pain perception have been reported in various contexts; however, their impact on postoperative pain intensity remains poorly understood, especially across different surgical procedures. Data from five hospitals were retrospectively analyzed, encompassing patients who underwent 10 distinct surgical procedures. Numeric rating scale scores were used to assess the worst postoperative pain intensity during the 24 h after surgery. The multivariate linear regression model analyzed the relationship between age and pain intensity. Subgroup analyses were performed according to sex and patient-controlled analgesia (PCA). This study included 41,187 patients. Among the surgeries studied, lumbar spine fusion (β = −0.155, *p* < 0.001) consistently and significantly exhibited a decrease in worst postoperative pain with increasing age. Similar trends were observed in cholecystectomy (β = −0.029, *p* < 0.001) and several other surgeries; however, the results were inconsistent across all analyses. Surgeries with higher percentages of PCA administration had lower median worst-pain scores. In conclusion, age may affect postoperative pain intensity after specific surgeries; however, a comprehensive understanding of the complex interplay between age, surgical intervention, and pain intensity is required. Pain management strategies should consider various factors, including age-related variations.

## 1. Introduction

Pain is a common and complex experience following surgical procedures that varies in intensity and duration across individuals and surgical interventions [1]. However, pain intensity on the first day after surgery is the highest in many surgical procedures [2,3]. Understanding the factors influencing postoperative pain on the first postoperative day is crucial for improving patient outcomes, optimizing pain management strategies, and enhancing overall patient satisfaction [4,5,6]. Pain management in the immediate postoperative period is critical because it can significantly affect recovery, patient comfort, and overall healthcare costs [7,8,9].

Among the various factors that can influence postoperative pain perception, age has been identified as a significant predictor [10,11,12]. However, in some studies, there were no differences in pain perception based on age [13,14]. However, the surgical incision, size of the surgical wound, method of anesthesia, and pain control differ according to the type of surgery. Therefore, it may be difficult to generalize the association between age and pain intensity. However, the results of these previous studies are limited [5,10,13,14].

This study investigated the association between age and pain intensity experienced on the first day after surgery in 10 distinct surgical procedures. The 10 surgeries under examination included arthroscopic rotator cuff repair (ARCR), cesarean section (C/sec), cholecystectomy, hip hemiarthroplasty, laparoscopic herniorrhaphy, open hernia repair, hysterectomy, lumbar spine fusion, total hip arthroplasty (THA), and total knee arthroplasty (TKA). By identifying the potential associations between age and pain intensity in various surgical contexts in five South Korean hospitals, we aimed to clarify age-related differences in postoperative pain perception, paving the way for tailored pain management strategies based on patient age and specific surgical procedures. The surgical types were determined based on previous studies; we extended our analysis to expand the scope of surgeries, encompassing obstetric and gynecologic surgeries—including cesarean sections and hysterectomies—and herniorrhaphies, a surgical procedure commonly performed on elderly patients.

## 2. Materials and Methods

### 2.1. Data Collection

The Clinical Research Ethics Committee of Chuncheon Sacred Heart Hospital, Hallym University, approved this retrospective cohort study. The need for informed consent was waived due to the study’s retrospective design. All data were collected from five hospitals at Hallym Medical Center through electronic medical records. Our dataset included patients’ baseline characteristics and records of anesthesia, surgery, laboratory examination, and prescription between 1 January 2013, and 30 June 2023.

### 2.2. Inclusion and Exclusion Criteria

This study included adult patients aged ≥ 18 who underwent one of the 10 surgical procedures between 1 January 2013, and 30 June 2023. The 10 surgical procedures were as follows: ARCR, C/sec, laparoscopic cholecystectomy, hip hemiarthroplasty, laparoscopic inguinal herniorrhaphy, open inguinal herniorrhaphy, hysterectomy, lumbar spine fusion, THA, and TKA. Surgery types were classified according to the Health Insurance Charge Code of the Republic of Korea (Appendix A). Laparoscopic surgery is classified based on surgical records because the codes for laparoscopic and open surgery are identical. 

This study excluded patients with cooperative surgery, missing data, or multiple surgeries.

### 2.3. Exposure and Primary and Secondary Outcomes

The exposure was the age (years) of the patients. The primary outcome was the worst pain intensity during the 24 h after surgery. Pain intensity was determined using a numeric rating scale (NRS) ranging from 0 (least) to 10 (worst). Secondary outcomes were the dose of opioids used during the postoperative 24 h. Opioids included morphine, oxycodone, meperidine, nalbuphine, tramadol, transdermal fentanyl patch, transdermal buprenorphine patch, fentanyl, and alfentanil. Because several types of opioids were included, the conversion dose was calculated based on morphine. The ratio of the conversion dose is presented in Appendix B.

### 2.4. Covariates

The covariates included patient characteristics, such as body mass index, comorbidities (hypertension, diabetes, tuberculosis, hepatitis, cardiac disease, cerebral vascular accident, cancer, hyperlipidemia, and sleep disorder), history of alcohol consumption, smoking, and medication (opioid, sleeping pills, and psychotropic drugs), and perioperative factors, such as physical status, emergency, anesthesia, surgery time, fluid balance, intraoperative monitoring (arterial and central venous pressures, Foley catheter, and Levin tube), and patient-controlled analgesia (route, adjuvant, and rate).

### 2.5. Statistics

Continuous data were expressed as medians and interquartile ranges, and categorical data were expressed as frequencies and percentages. We used a locally estimated scatterplot smoothing line to fit a smooth curve through the scatterplot of age and NRS. Visualizing the relationship between age and NRS as an optimal-fit curve makes it easier for readers to understand.

Considering the covariates, we analyzed the adjusted association between age and worst pain at 24 h postoperatively using linear regression. The partial R^2^ between age and NRS score was also calculated. For the subgroup analysis, we analyzed the adjusted association between age and worst pain 24 h postoperatively depending on sex and patient-controlled analgesia (PCA) administration.

Using Bonferroni correction, a *p*-value ≤ 0.005 was considered statistically significant. SPSS version 26.0 (IBM, Armonk, NY, USA) was used for all analyses, and graphs were drawn using R version 4.2.3 (R Foundation for Statistical Computing, www.r-project.org [accessed on 10 September 2023]).

## 3. Results

### 3.1. Patient Characteristics

**Figure 1:**
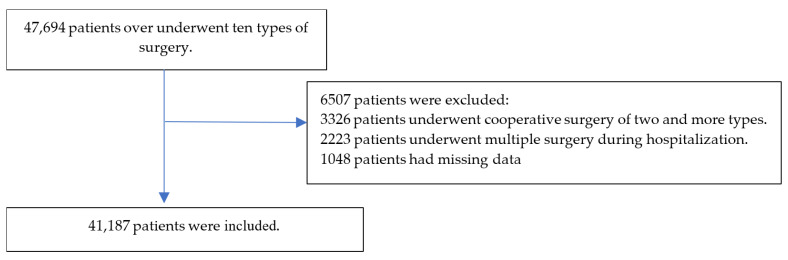
Flow chart of participant selection.

This study enrolled 47,694 patients aged ≥ 18 who underwent 10 types of surgical procedures between January 2013 and June 2023. A total of 6507 patients were excluded, and 41,187 patients were included in this study (Figure 1). The number, characteristics, and perioperative data according to surgery type are summarized in Table 1.

### 3.2. Pain Intensity and Dose of Opioid

The median worst pain intensity according to surgery ranged from 3 to 5 on the NRS. Surgeries with a median NRS score of ≥4 included ARCR, cholecystectomy, laparoscopic herniorrhaphy, open herniorrhaphy, lumbar spine fusion, and TKA.

The median opioid dose administered during surgery ranged from 0 to 28.8 mg, depending on the surgery. In herniorrhaphy, ≥75% of the patients did not use opioids. Details of pain intensity and opioid dose administered during the 24 h after surgery are summarized in Table 2.

### 3.3. Effects of Age on Worst Pain during the 24 h after Surgery

**Figure 2:**
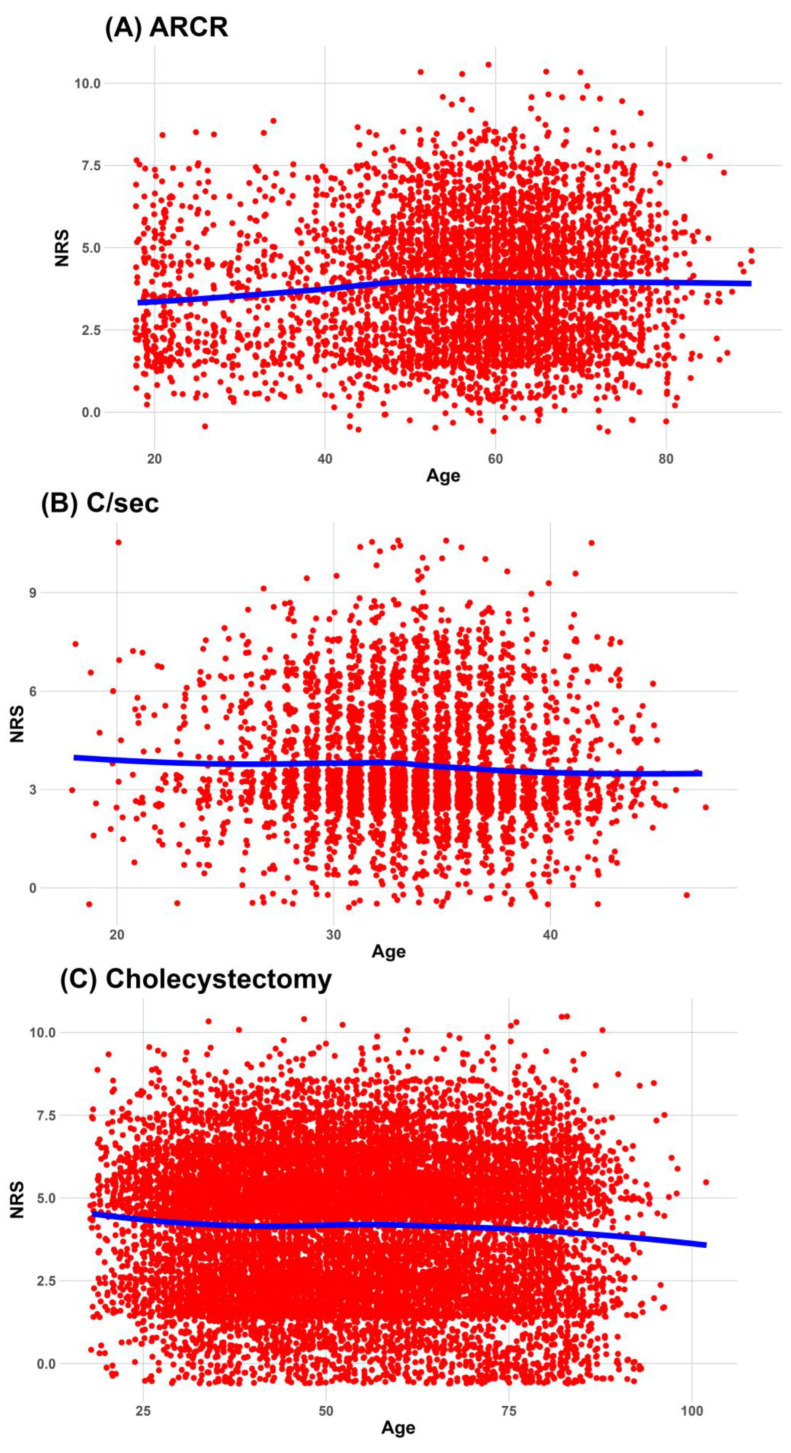

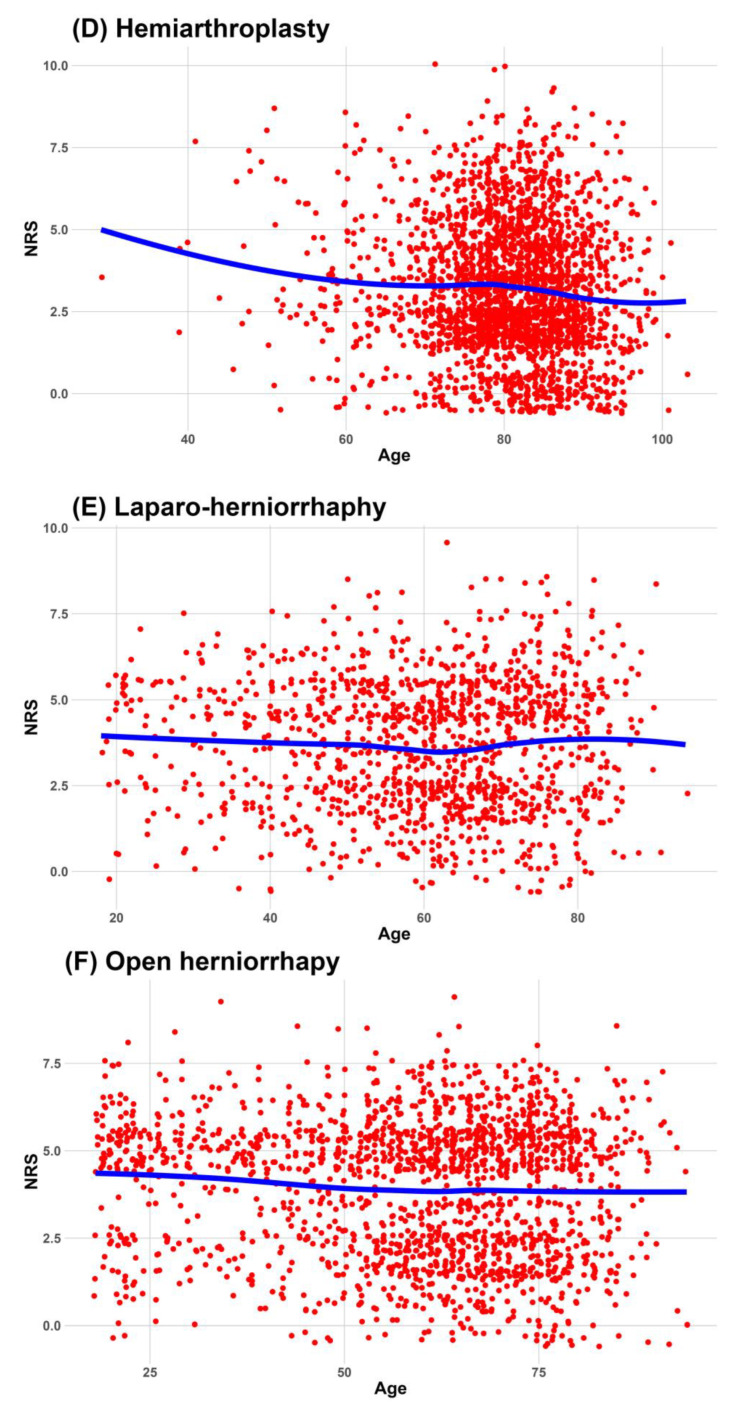

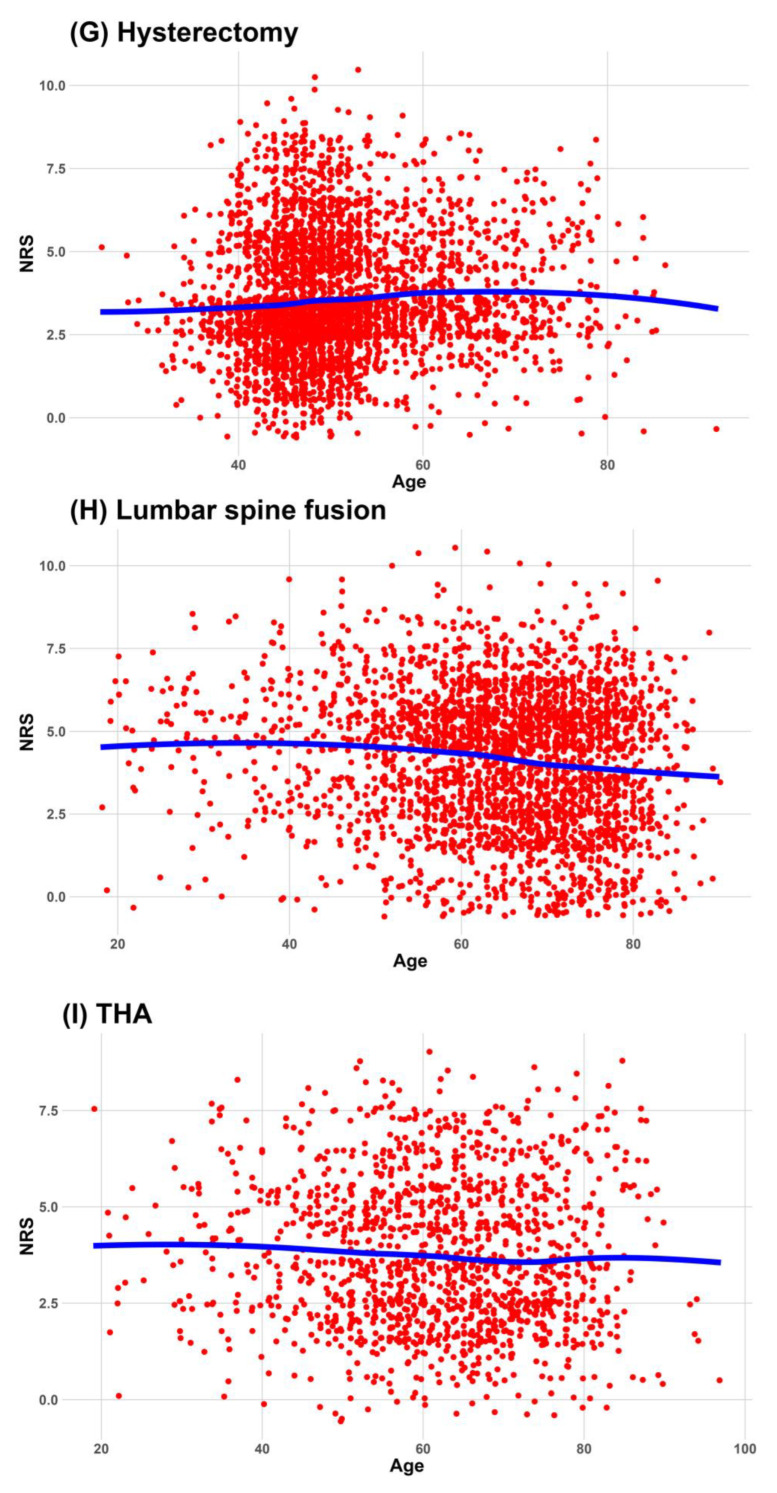

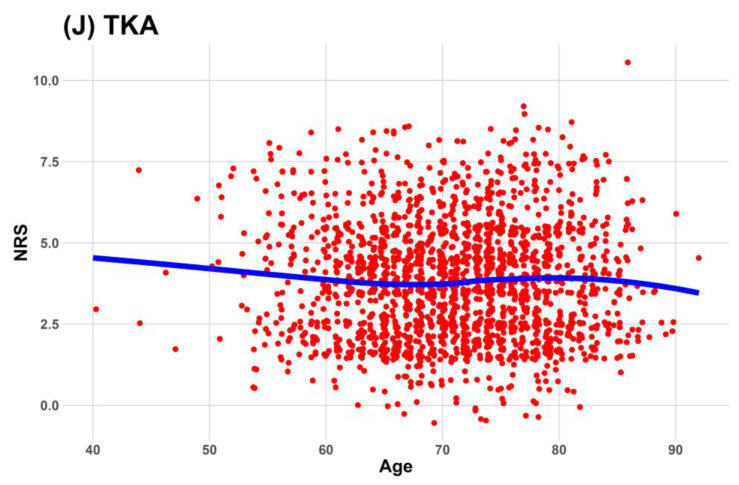
Association between the worst pain during 24 h after surgery and age. Red dots are scatterplot of Age and NRS. Blue line is estimated scatterplot smoothing line. (**A**) association between Age and NRS in ARCR, (**B**) association between Age and NRS in C/sec, (**C**) association between Age and NRS in cholecystectomy, (**D**) association between Age and NRS in hemiarthroplasty, (**E**) association between Age and NRS in laparo-herniorrhaphy, (**F**) association between Age and NRS in open-herniorrhaphy, (**G**) association between Age and NRS in hysterectomy, (**H**) association between Age and NRS in lumbar spine fusion, (**I**) association between Age and NRS in THA, (**J**) association between Age and NRS in TKA. ARCR, arthroscopic rotator cuff repair; C/sec, cesarean section; NRS, numeric rating scale; THA, total hip arthroplasty; TKA, total knee arthroplasty.

The association between the worst pain score during the 24 h after surgery and age is summarized in Figure 2. In most surgeries, locally estimated scatterplot smoothing lines did not show an age-related decrease in the worst pain score 24 h after surgery. Only after hip hemiarthroplasty does the worst pain score decrease with age and stabilize at approximately 70 years of age.

However, for seven types of surgery—those other than C/s, laparoscopic herniorrhaphy, and open herniorrhaphy—the worst pain scores decreased significantly with age when other factors were adjusted. Details of the association between age and worst pain during the 24 h after surgery are summarized in Table 3.

### 3.4. Association between Age and Worst Pain According to Sex and PCA Administration 24 h after Surgery

Considering the surgical characteristics, C/sec and hysterectomy were excluded from the analysis based on sex. The worst pain scores for cholecystectomy and lumbar spine fusion decreased significantly in males and females. Details of the association between age and worst pain according to sex during the 24 h after surgery are summarized in Table 4.

The number of patients who received PCA was 29,388. The percentage of patients who received PCA in ARCR, C/sec, hip hemiarthroplasty, hysterectomy, lumbar spine fusion, TKA, and THA was over 80%; however, that of herniorrhaphy was 10–20%. Except for ARCA and hysterectomy, the worst pain score significantly decreased with age in patients who received PCA. Details of the association between age and the worst pain during the 24 h after surgery in patients who received PCA are summarized in Table 5.

### 3.5. Effects of Age on the Administered Opioid Dose during the 24 h after Surgery

**Figure 3:**
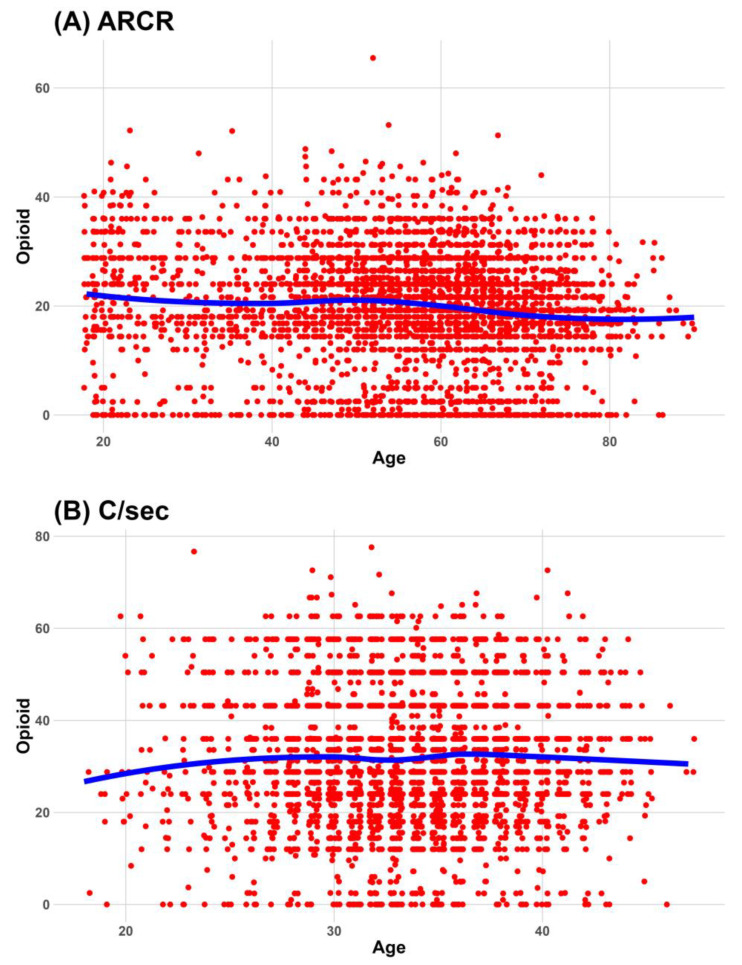

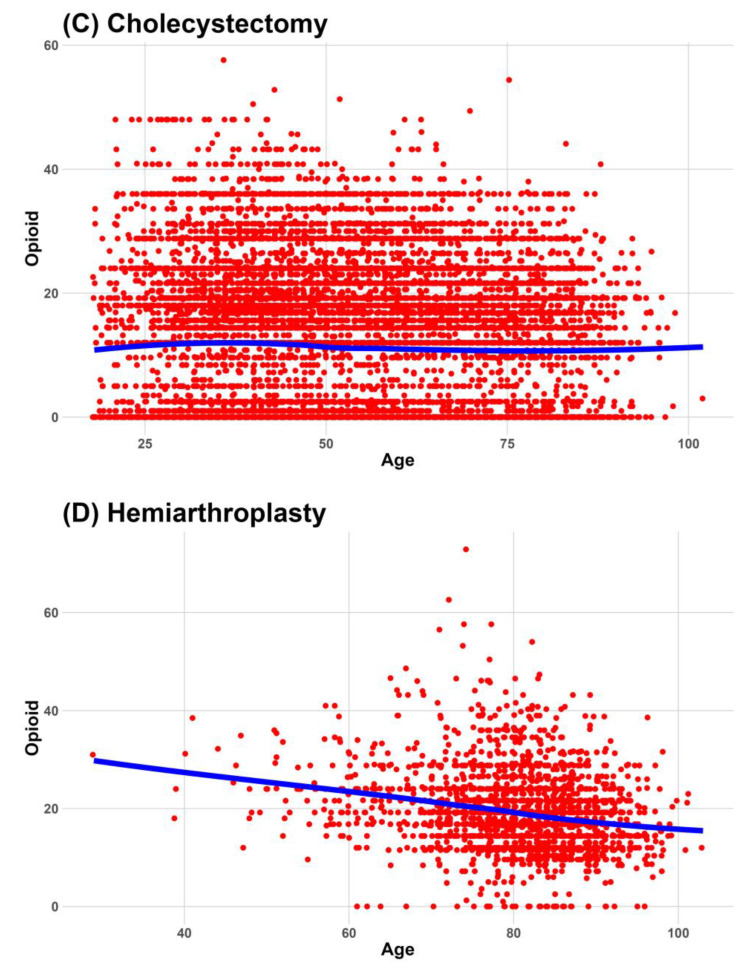

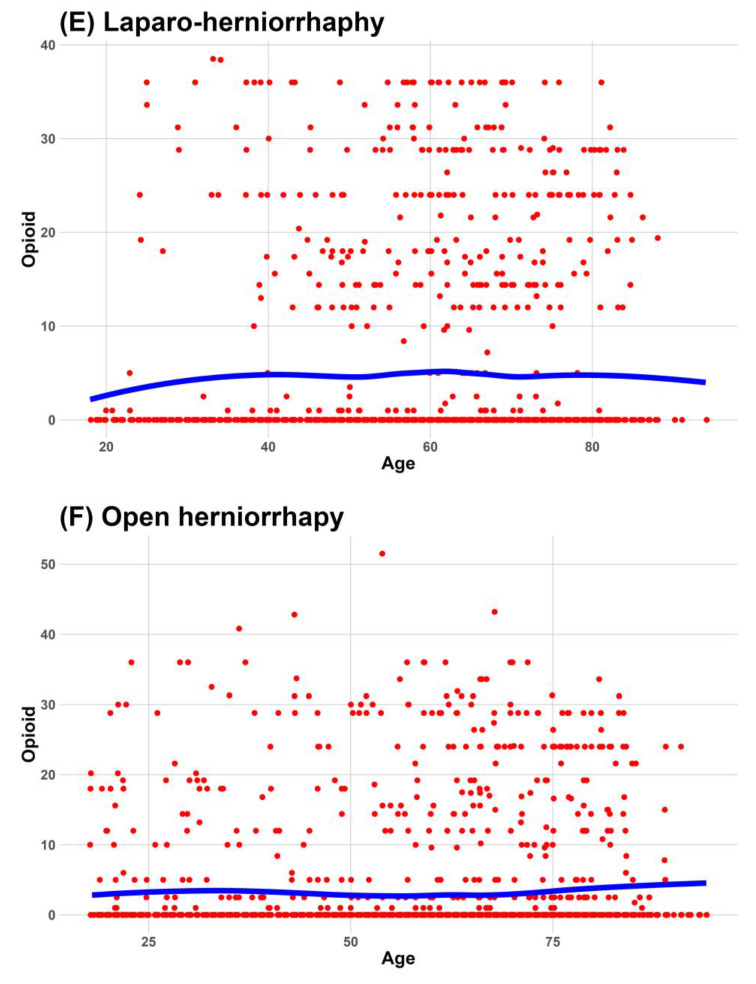

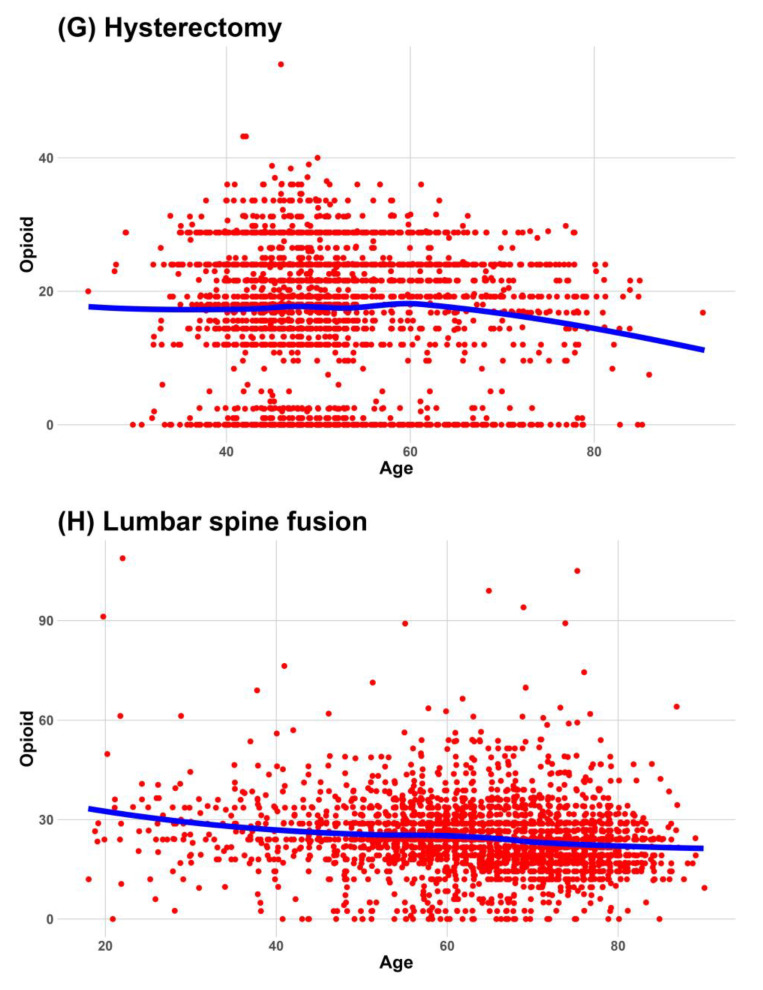

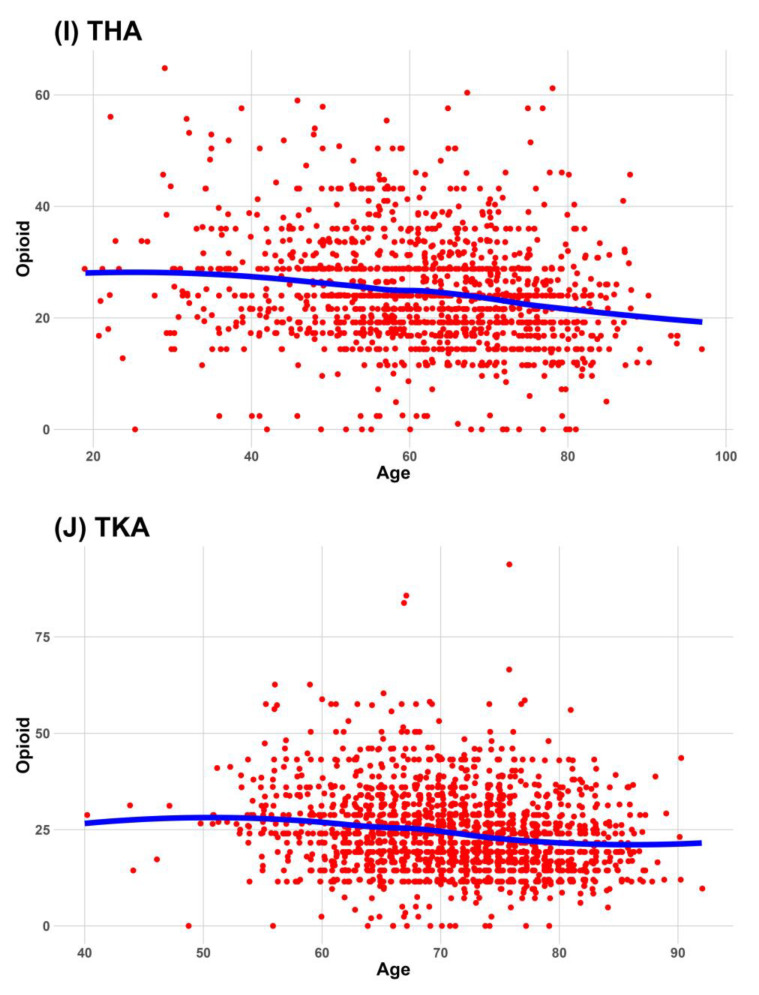
Association between the administered opioid dose during 24 h after surgery and age. Red dots are scatterplot of Age and administered opioid dose during 24 h after surgery. Blue line is estimated scatterplot smoothing line. (**A**) association between Age and administered opioid dose during 24 h after surgery in ARCR, (**B**) association between Age and administered opioid dose during 24 h after surgery in C/sec, (**C**) association between Age and administered opioid dose during 24 h after surgery in cholecystectomy, (**D**) association between Age and administered opioid dose during 24 h after surgery in hemiarthroplasty, (**E**) association between Age and administered opioid dose during 24 h after surgery in laparo-herniorrhaphy, (**F**) association between Age and administered opioid dose during 24 h after surgery in open-herniorrhaphy, (**G**) association between Age and administered opioid dose during 24 h after surgery in hysterectomy, (**H**) association between Age and administered opioid dose during 24 h after surgery in lumbar spine fusion, (**I**) association between Age and administered opioid dose during 24 h after surgery in THA, (**J**) association between Age and administered opioid dose during 24 h after surgery in TKA. ARCR, arthroscopic rotator cuff repair; C/sec, cesarean section; NRS, numeric rating scale; THA, total hip arthroplasty; TKA, total knee arthroplasty. ARCR, arthroscopic rotator cuff repair; C/sec, cesarean section; THA, total hip arthroplasty; TKA, total knee arthroplasty.

Locally estimated scatterplot smoothing lines show an age-related decrease in the administered dose of opioids during the 24 h after surgery in several surgeries, such as hip hemiarthroplasty, hysterectomy, lumbar spine fusion, THA, and TKA. The association between the administered dose of opioids during the 24 h after surgery and age is summarized in Figure 3.

However, for six types of surgery, including ARCR, cholecystectomy, hip hemiarthroplasty, lumbar spine fusion, THA, and TKA, the administered dose of opioids was significantly reduced with age when other factors were adjusted. Details of the association between age and the administered dose of opioids during the 24 h after surgery are summarized in Table 6.

## 4. Discussion

This study investigated the relationship between age and the worst postoperative pain during the 24 h after surgery in 10 surgical procedures. The worst pain associated with cholecystectomy and lumbar spine fusion decreased consistently and significantly with increasing age in all analyses, including the subgroup analysis. These were also included in six surgeries (ARCR, cholecystectomy, hip hemiarthroplasty, lumbar spine fusion, THA, and TKA), which decreased the administered dose of opioids with age. However, considering a 2.4 unit reduction in the NRS is required for clinically meaningful pain relief in moderate or severe pain [15], only lumbar spine fusion showed clinical and statistical decreases in pain with age consistently and significantly in several analyses.

Jacqueline et al. reported an association between postoperative pain and age using an international database of hip replacement, knee replacement, spinal fusion, and laparoscopic surgery [12]. Their study included various pain measurements such as worst pain, pain associated with moving, breathing/coughing, and sleeping, and the worst postoperative pain decreased with increasing age in all four types of surgeries. Our study included the types of surgeries used in their study; however, there were several differences between them. Our analyses did not all consistently show a decrease in worst pain with increasing age in hip hemiarthroplasty, THA, TKA, or laparoscopic cholecystectomy, except for lumbar spine fusion. Their worst NRS scores were generally higher than those in our study. The patients in our study who underwent lumbar spinal fusion, THA, TKA, or hip hemiarthroplasty had a worst NRS score approximately 1–2 units lower than others in their study. Considering their higher NRS scores, information on postoperative pain control modalities was insufficient. TKA, THA, hemiarthroplasty, and lumbar spinal fusion have long skin incisions and comparable surgical wounds, and several postoperative pain control modalities, including PCA, can be applied. Therefore, it is difficult to generalize the results without knowing how pain was controlled.

However, two studies reported an association between age and postoperative pain in patients using analgesic methods. Koh JC et al. investigated postoperative pain in young (age 20–39 years) and older adult (age ≥ 70 years) patients with patient-controlled epidural analgesia application [11]. Although they did not analyze the type of surgery, the mean NRS score for pain intensity within 24 h was 3–5. Young patients had significantly more severe pain than older adult patients 6 h after surgery. Barrington et al. studied postoperative pain after TKA in patients who received local analgesic injections [10]. They investigated average pain intensity using the visual analog scale (VAS) and age-reduced pain intensity using multivariate regression analysis for overall average VAS pain scores. Our study also showed that increasing age decreased pain intensity in patients who received PCA. However, the two previous studies did not investigate the worst pain separately, and the overall pain intensity of patients with pain control was not high. One study showed that the differences in NRS scores between old and young patients were <1 unit [11]. In our study, the median worst pain intensity in patients who received PCA was <4 in several surgeries. In patients with low pain intensity, the clinical effect of age on pain intensity was not statistically significant.

In our study, we found that the median NRS worst pain score was the same in all patients and in patients who received PCA in seven surgeries that had a high percentage of PCA administration, such as ARCR, C/sec, hip hemiarthroplasty, hysterectomy, lumbar spine fusion, THA, and TKA. In contrast, in surgeries with a low percentage of PCA administration, such as cholecystectomy and herniorrhaphy, the median NRS worst pain score in patients who received PCA was lower than that in all patients. These results were consistent with the order of the administered median doses of opioids according to surgery. According to the administered opioid dose, the top seven surgeries were those that had the same median NRS for patients with and without PCA, while the bottom three surgeries were those that had different median NRS for patients with and without PCA. However, to clarify the association between opioids and the worst pain, further studies that consider the adverse effects of high doses of opioids are needed.

The effects of aging on pain intensity should be considered based on several factors. Aging is associated with anatomical and neurochemical changes [16] affecting pain perception. Aging is associated with increased pain thresholds [17,18] and reflects reduced pain sensitivity [19]. It has been reported that pain intensity in chronic pain does not vary with age [20,21,22]; however, acute pain can be caused by damage, such as surgery, and is related to skeletal muscle spasms and sympathetic nervous system activation [23]. An experimental study showed that aging affects the fast initial pain response but does not affect the sustained pain response [24]. The effect of age on pain intensity may differ according to surgery as pain threshold differs according to the size of the surgery and the peripheral cutaneous or visceral sites [17].

The relationship observed between age and postoperative pain intensity in various surgical procedures should be considered the potential role of pharmacokinetics, especially in the context of opioids. Opioids are commonly used analgesics for postoperative pain management, and their pharmacokinetic profiles can vary with age, which could potentially impact pain outcomes in the elderly [25]. Elderly patients often show a decrease in hepatic enzyme activity, which can lead to an extended half-life of opioids [26], which can impact pain relief. Aging is also associated with changes in receptor sensitivity, including opioid receptors, so elderly patients may respond differently to opioids than younger patients [27,28]. Given these pharmacokinetic considerations, the variable effects of age on postoperative pain intensity observed in this study may be partly due to differences in the way opioids are metabolized and utilized in elderly patients compared to younger individuals.

The strength of this study is that it focused on 10 types of surgeries, which may represent the various spectra of surgical procedures, and investigated patients using pain control modalities, such as PCA. However, this study has some limitations:

The present study used the NRS to assess pain intensity. Although the NRS is a commonly used tool, it is subjective and may not capture the full complexity of pain experiences. Nonetheless, it provides a structured format, has good sensitivity, and generates data that can be statistically analyzed for audit purposes compared to the VAS, which may reduce variability in interpretation [29,30,31].

This study focused on postoperative pain within 24 h after surgery. Pain experiences might change over an extended period, and considering pain trajectories beyond the first 24 h could provide additional insights.

This study acknowledges that various pain control modalities may have been used and that some data may be heterogeneous, except for PCA, which could have influenced pain intensity. Recently, multimodal analgesic methods are used, even in minor surgeries such as laparoscopic cholecystectomy and inguinal hernia repair [29,30]. Specific details regarding the types of analgesic methods and their dosages, except for included factors, are yet to be discussed, making it challenging to understand their full impact on the results.

While our intention was to assemble a diverse dataset to bolster the generalizability of our results, this study may be susceptible to potential biases stemming from variations in hospital protocols, patient populations, and data-collection methodologies. Moreover, these variations may potentially influence the observed outcomes.

In conclusion, this study provided valuable insights into the relationship between patient age and postoperative pain during various surgical procedures. Although age influences postoperative pain intensity in some surgeries, the findings are inconsistent across all procedures. They can differ according to the type of surgery and PCA administration. These findings suggest that the impact of pain control modalities emphasizes the importance of personalized pain management approaches. Future research should address the limitations of this study and explore additional factors that might influence postoperative pain to enable better pain management practices and improve patient outcomes.

## Figures and Tables

**Table 1:** Characteristics and perioperative patient data.

	ARCR (*n* = 4875)	C/Sec (*n* = 4146)	CC (*n* = 15,947)	HHA (*n* = 2697)	LH (*n* = 1322)	OH (*n* = 1822)	HyE (*n* = 3977)	LSF (*n* = 3024)	THA (*n* = 1471)	TKA (*n*= 1906)
Age (year)	59 (50–66)	34 (31–37)	52 (40–65)	82 (77–86)	63 (51–72)	63 (49–72)	49 (45–54)	66 (58–73)	63 (54–71)	71 (66–76)
Female	2180 (44.7)	4146 (100)	8346 (52.3)	2088 (77.4)	78 (5.9)	148 (8.1)	3977 (100)	1668 (55.2)	697 (47.4)	1479 (77.6)
BMI (kg/m^2^)	25 (22.9–27.2)	27.5 (25.1–30.7)	25 (22.7–27.5)	22.1 (19.6–24.5)	23.6 (21.9–25.4)	23.7 (21.8–25.4)	24.3 (22.2–26.9)	24.9 (22.6–27.2)	23.7 (21.4–26.4)	26.4 (24.1–28.9)
HTN	1436 (29.5)	100 (2.4)	4264 (26.7)	1373 (50.9)	415 (31.4)	509 (27.9)	784 (19.7)	1372 (45.4)	490 (33.3)	1016 (53.3)
DM	616 (12.6)	226 (5.5)	2063 (12.9)	667 (24.7)	123 (9.3)	157 (8.6)	272 (6.8)	644 (21.3)	209 (14.2)	426 (22.4)
TB	46 (0.9)	39 (0.9)	168 (1.1)	48 (1.8)	24 (1.8)	31 (1.7)	35 (0.9)	43 (1.4)	21 (1.4)	18 (0.9)
Hepatitis	68 (1.4)	52 (1.3)	218 (1.4)	16 (0.6)	14 (1.1)	29 (1.6)	59 (1.5)	37 (1.2)	18 (1.2)	14 (0.7)
Cardiac disease	151 (3.1)	11 (0.3)	453 (2.8)	213 (7.9)	51 (3.9)	71 (3.9)	47 (1.2)	179 (5.9)	64 (4.4)	117 (6.1)
CVA	56 (1.1)	2 (0)	230 (1.4)	149 (5.5)	16 (1.2)	28 (1.5)	20 (0.5)	58 (1.9)	31 (2.1)	43 (2.3)
Cancer	103 (2.1)	36 (0.9)	451 (2.8)	169 (6.3)	32 (2.4)	93 (5.1)	156 (3.9)	132 (4.4)	53 (3.6)	74 (3.9)
Hyperlipidemia	651 (13.4)	13 (0.3)	1655 (10.4)	242 (9)	107 (8.1)	93 (5.1)	387 (9.7)	415 (13.7)	169 (11.5)	340 (17.8)
Alcohol	1569 (32.2)	28 (0.7)	4535 (28.4)	133 (4.9)	440 (33.3)	591 (32.4)	416 (10.5)	603 (19.9)	414 (28.1)	195 (10.2)
Smoking	861 (17.7)	34 (0.8)	2646 (16.6)	126 (4.7)	240 (18.2)	350 (19.2)	154 (3.9)	416 (13.8)	340 (23.1)	85 (4.5)
Sleep disorder	152 (3.1)	15 (0.4)	418 (2.6)	183 (6.8)	14 (1.1)	22 (1.2)	74 (1.9)	160 (5.3)	63 (4.3)	89 (4.7)
Sleep pills	114 (2.3)	2 (0)	326 (2)	186 (6.9)	11 (0.8)	21 (1.2)	46 (1.2)	118 (3.9)	64 (4.4)	79 (4.1)
Psychotropic drug	57 (1.2)	11 (0.3)	187 (1.2)	91 (3.4)	3 (0.2)	13 (0.7)	39 (1)	53 (1.8)	34 (2.3)	39 (2)
ASA PS (grade)	2 (1–2)	2 (1–2)	2 (1–2)	3 (2–3)	2 (1–2)	2 (1–2)	2 (1–2)	2 (2–3)	2 (2–3)	2 (2–3)
EM	54 (1.1)	1886 (45.5)	2681 (16.8)	312 (11.6)	25 (1.9)	73 (4)	42 (1.1)	158 (5.2)	67 (4.6)	17 (0.9)
GA	4870 (99.9)	1627 (39.2)	15,946 (100)	1763 (65.4)	1322 (100)	1134 (62.2)	3975 (99.9)	3024 (100)	922 (62.7)	901 (47.3)
OP time (hour)	1.3 (1–1.6)	1 (0.8–1.2)	0.8 (0.6–1)	1.2 (0.9–1.5)	1 (0.8–1.3)	0.8 (0.5–1.1)	2 (1.6–2.5)	3.5 (2.8–4.6)	1.6 (1.3–2)	1.8 (1.4–2.3)
Fluid balance (L)	0.5 (0.4–0.6)	0.7 (0.4–1)	0.3 (0.2–0.5)	0.6 (0.3–0.9)	0.4 (0.3–0.5)	0.4 (0.2–0.5)	0.5 (0.3–0.8)	1 (0.6–1.5)	0.7 (0.3–1.1)	0.4 (0.1–0.7)
A-line	3084 (63.3)	363 (8.8)	2949 (18.5)	2624 (97.3)	215 (16.3)	151 (8.3)	1293 (32.5)	2951 (97.6)	1329 (90.3)	1435 (75.3)
C-line	12 (0.2)	44 (1.1)	94 (0.6)	663 (24.6)	2 (0.2)	6 (0.3)	59 (1.5)	920 (30.4)	311 (21.1)	221 (11.6)
Foley	302 (6.2)	4041 (97.5)	1125 (7.1)	2415 (89.5)	19 (1.4)	30 (1.6)	3829 (96.3)	2978 (98.5)	1192 (81)	1516 (79.5)
L-tube	2 (0)	3 (0.1)	102 (0.6)	7 (0.3)	3 (0.2)	4 (0.2)	4 (0.1)	5 (0.2)	1 (0.1)	2 (0.1)
Opioids Hx.	420 (8.6)	8 (0.2)	516 (3.2)	159 (5.9)	16 (1.2)	41 (2.3)	45 (1.1)	723 (23.9)	200 (13.6)	248 (13)
NSAIDs HX.	1423 (29.2)	110 (2.7)	1677 (10.5)	243 (9)	63 (4.8)	90 (4.9)	281 (7.1)	998 (33)	325 (22.1)	559 (29.3)
IV-PCA	4167 (85.5)	2191 (52.8)	8610 (54)	2029 (75.2)	265 (20)	231 (12.7)	3179 (79.9)	2937 (97.1)	995 (67.6)	1055 (55.4)
PCA adjuvant	1521 (31.2)	972 (23.4)	1554 (9.7)	364 (13.5)	97 (7.3)	75 (4.1)	764 (19.2)	652 (21.6)	276 (18.8)	300 (15.7)
PCA flow rate (mg/h)	1.6 (1.2–2)	2.4 (2–3.6)	1 (0–1.6)	1.4 (1–1.8)	0 (0–0)	0 (0–0)	1.6 (1–2)	1.8 (1.4–2)	1.9 (1.5–2.4)	1.8 (1.2–2)

The data have been presented in terms of the median and interquartile range or as numerical counts and corresponding percentages. The numeric values provided without specified units correspond to the patient count A-line, arterial cannulation line; ARCR, arthroscopic rotator cuff repair; ASA PS; American Society of Anesthesiologists physical status; BMI, body mass index; CC, cholecystectomy; C-line, central venous line; L-tube, Levin tube; C/sec, cesarean section; CVA, cerebral vascular accident; DM, diabetes; EM, emergency; GA, general anesthesia; HHA, hip hemi-arthroplasty; HTN, hypertension; HyE, hysterectomy; Hx. History; LH, laparoscopic herniorrhaphy; LSF, lumbar spine fusion; NSAID Hx., taking non-steroidal anti-inflammatory drugs for 30 days before hospitalization, OH, open herniorrhaphy; OP, operation; Opioid Hx., taking opioids for 30 days before hospitalization; PCA, patient-controlled analgesia; TB, tuberculosis; THA, total hip arthroplasty; TKA, total knee arthroplasty.

**Table 2:** Pain intensity and administered opioid dose during 24 h after surgery.

	Number of Patients	Worst NRS during Postoperative 24 h	Least NRS during Postoperative 24 h	Dose of Opioid during Postoperative 24 h, mg (IQR, Minimum, and Maximum)
ARCR	4725	4 (2–5)	1 (1–2)	21.6 (15.6–25.2, 0, 65.5)
C/sec	4105	3 (3–5)	2 (1–3)	28.8 (24–43.2, 0, 77.6)
Cholecystectomy	13,151	5 (2–6)	1 (0–2)	12 (0–19.2, 0, 57.6)
Hip hemiarthroplasty	2581	3 (2–5)	1 (0–2)	18 (14.4–24, 0, 72.9)
Laparo-herniorrhaphy	344	4 (2–5)	0 (0–1)	0 (0–0, 0, 38.5)
Open herniorrhaphy	783	5 (2–5)	1 (0–2)	0 (0–0, 0, 51.5)
Hysterectomy	3938	3 (3–5)	2 (1–3)	19.4 (12–24, 0, 54)
Lumbar spine fusion	2872	5 (3–6)	1 (0–2)	24 (19.2–28.8, 0, 108)
THA	1446	3 (2–5)	2 (1–2)	24 (19.2–28.8, 0, 64.8)
TKA	1888	4 (2–5)	2 (1–2)	24 (16.8–28.8, 0, 93.8)

ARCR, arthroscopic rotator cuff repair; C/sec, cesarean section; IQR, interquartile range; NRS, numeric rating scale; PCA, patient-controlled analgesia; THA, total hip arthroplasty; TKA, total knee arthroplasty.

**Table 3:** Linear regression of the relationship between age and the worst pain during 24 h after surgery for 10 types of surgeries.

Type of Surgery	Partial R^2^	β ± SE	*p* Value	Numeric Rating Scale Score Difference over a Time Span of 30 Years
ARCR (*n* = 4875)	−0.059	−0.077 ± 0.008	<0.001	2.3
C/sec (*n* = 4146)	−0.014	−0.014 ± 0.027	0.115	0.4
Cholecystectomy (*n* = 15,947)	−0.021	−0.029 ± 0.003	<0.001	0.9
Hip hemiarthroplasty (*n* = 2697)	−0.143	−0.153 ± 0.016	<0.001	4.6
Laparoscopic herniorrhaphy (*n* = 1322)	−0.02	−0.026 ± 0.008	0.028	0.8
Open herniorrhaphy (*n* = 1822)	−0.013	−0.016 ± 0.005	0.15	0.5
Hysterectomy (*n* = 3977)	−0.021	−0.024 ± 0.009	0.004	0.7
Lumbar spine fusion (*n* = 3024)	−0.126	−0.155 ± 0.017	<0.001	4.6
THA (*n* = 1471)	−0.087	−0.105 ± 0.019	<0.001	3.1
TKA (*n* = 1906)	−0.09	−0.101 ± 0.03	<0.001	3

ARCR, arthroscopic rotator cuff repair; C/sec, cesarean section; SE, standard error; THA, total hip arthroplasty; TKA, total knee arthroplasty.

**Table 4:** Linear regression of the relationship between age and the worst pain during 24 h after surgery according to sex for eight types of surgeries.

	Male			Female		
Type of Surgery (Male/Female)	Partial R^2^	β ± SE	*p* Value	Partial R^2^	β ± SE	*p* Value
ARCR (2695/2180)	0.047	0.06 ± 0.003	0.016	−0.021	−0.025 ± 0.005	0.322
Cholecystectomy (7061/8346)	−0.055	−0.073 ± 0.002	<0.001	−0.065	−0.088 ± 0.002	<0.001
Hip hemiarthroplasty (609/2088)	−0.055	−0.063 ± 0.009	0.17	−0.052	−0.054 ± 0.006	0.015
Laparoscopic herniorrhaphy (1244/78)	−0.104	−0.136 ± 0.008	0.049	−0.089	−0.162 ± 0.059	0.772
Open-herniorrhaphy (1674/148)	−0.112	−0.147 ± 0.005	0.002	0.066	0.142 ± 0.03	0.617
Lumbar spine fusion (1356/1668)	−0.116	−0.148 ± 0.005	<0.001	−0.127	−0.15 ± 0.005	<0.001
THA (774/697)	−0.052	−0.062 ± 0.006	0.124	−0.013	−0.015 ± 0.006	0.731
TKA (427/1479)	−0.032	−0.037 ± 0.012	0.498	−0.003	−0.004 ± 0.006	0.9

ARCR, arthroscopic rotator cuff repair; SE, standard error; THA, total hip arthroplasty; TKA, total knee arthroplasty.

**Table 5:** Worst NRS and linear regression of the relationship between age and the worst pain in patients with PCA application during 24 h after surgery.

Type of Surgery (*n*)	Partial R^2^	β ± SE	*p* Value	Worst NRS during Postoperative 24 h in Patients with PCA
ARCR (*n* = 4167)	0.021	0.028 ± 0.003	0.028	4 (2–5)
C/sec (*n* = 4013)	−0.04	−0.041 ± 0.006	<0.001	3 (3–5)
Cholecystectomy (*n* = 8610)	−0.057	−0.077 ± 0.002	<0.001	3 (2–5)
Hip hemiarthroplasty (*n* = 2650)	−0.059	−0.063 ± 0.005	<0.001	3 (2–5)
Laparoscopic herniorrhaphy (*n* = 265)	−0.012	−0.016 ± 0.009	<0.001	2 (2–3)
Open herniorrhaphy (*n* = 233)	−0.099	−0.144 ± 0.009	<0.001	3 (2–5)
Hysterectomy (*n* = 3183)	0.061	0.07 ± 0.004	0.07	3 (2–5)
Lumbar spine fusion (*n* = 2937)	−0.119	−0.146 ± 0.004	<0.001	5 (3–6)
THA (*n* = 1448)	−0.028	−0.033 ± 0.004	<0.001	3 (2–5)
TKA (*n* = 1882)	−0.006	−0.007 ± 0.006	<0.001	4 (2–5)

ARCR, arthroscopic rotator cuff repair; C/sec, cesarean section; NRS, numeric rating scale; PCA, patient-controlled analgesia; SE, standard error; THA, total hip arthroplasty; TKA, total knee arthroplasty.

**Table 6:** Linear regression of the relationship between age and the administered opioid dose during 24 h after surgery for ten types of surgeries.

Type of Surgery (*n*)	Partial R^2^	β ± SE	*p* Value
ARCR (*n* = 4875)	−0.064	−0.083 ± 0.009	<0.001
C/sec (*n* = 4146)	−0.012	−0.013 ± 0.027	0.149
Cholecystectomy (*n* = 15,947)	−0.023	−0.031 ± 0.004	<0.001
Hip hemiarthroplasty (*n* = 2697)	−0.14	−0.15 ± 0.017	<0.001
Laparoscopic herniorrhaphy (*n* = 1322)	−0.042	−0.055 ± 0.022	0.026
Open herniorrhaphy (*n* = 1822)	−0.028	−0.037 ± 0.01	0.029
Hysterectomy (*n* = 3977)	−0.019	−0.021 ± 0.009	0.009
Lumbar spine fusion (*n* = 3024)	−0.129	−0.158 ± 0.017	<0.001
THA (*n* = 1471)	−0.086	−0.103 ± 0.019	<0.001
TKA (*n* = 1906)	−0.087	−0.098 ± 0.03	<0.001

ARCR, arthroscopic rotator cuff repair; C/sec, cesarean section; SE, standard error; THA, total hip arthroplasty; TKA, total knee arthroplasty.

## Data Availability

Restrictions apply to data availability. Data were obtained from the Hallym Medical Center and are available from its clinical data warehouse with permission from the Hallym Medical Center.

## Appendix A. Surgery-Specific Codes for Determining the Type of Surgery (https://www.koicd.kr/main.do, accessed on 24 July 2023)

**Type of Surgery**	**Code**
ARCR	N0936, N0937, N0938,
Cesarean Section	R4514A, R4514J, R4516, R4517, R4517A, R4518, R4518A, R4519, R4519A, R4520, R4520A
Laparoscopic cholecystectomy	Q7380, Q7380G
Lumbar spine Fusion	N0466, N0469, N1460, N1466, N1469, N2470
Hip Hemiarthroplasty	N0715, N0715A, N1715, N1715A, N1725, N2710, N2710A, N4710, N4710A
Inguinal Herniorrhaphy	Q2753, Q2753G, Q2754G, Q2755, Q2755G, Q2756, Q2756G
Hysterectomy	R0141, R0142, R4143, R4144, R4147, R4148
THA	N0711, N0711A, N0711R, N1711, N1711A, N1721, N2070, N2070A, N3710, N3710A, N3720
TKA	N2072, N2077, N3712, N3717, N3722, N3727
ARCR, arthroscopic rotator cuff repair; THA, total hip arthroplasty; TKA, total knee arthroplasty.	

## Appendix B. Opioid Equivalent Dose for Opioid Dosage Calculation

**Drugs**	
Morphine	10 mg
Oxycodone	10 mg
Meperidine	100 mg
Nalbuphine	10 mg
Tramadol	100 mg
Transdermal Fentanyl patch	12.5 mcg/h
Transdermal Buprenorphine patch	10 mcg/h
Fentanyl	0.1 mg
Alfentanil	1 mg
Sufentanil	0.01 mg
